# The Energy to Smoke: Examining the Longitudinal Association between Beverage Consumption and Smoking and Vaping Behaviours among Youth in the COMPASS Study

**DOI:** 10.3390/ijerph18083864

**Published:** 2021-04-07

**Authors:** Matthew J. Fagan, Katie M. Di Sebastiano, Wei Qian, Scott T. Leatherdale, Guy Faulkner

**Affiliations:** 1 Population Physical Activity Laboratory, School of Kinesiology, University of British Columbia, Vancouver, BC V6T 1Z4, Canada; katie.disebastiano@ubc.ca (K.M.D.S.); guy.faulkner@ubc.ca (G.F.); 2 School of Public Health and Health Systems, University of Waterloo, Waterloo, ON N2L 3G1, Canada; wei.qian@uwaterloo.ca (W.Q.); sleatherdale@uwaterloo.ca (S.T.L.)

**Keywords:** adolescence, high-energy drink, sugar-sweetened beverages, caffeine, smoking, vaping, longitudinal

## Abstract

This study examined the longitudinal association between changes in sugar-sweetened and/or caffeinated beverage consumption and smoking/vaping behaviour among Canadian adolescents. Using longitudinal data from the COMPASS study (2015/16 to 2017/18), four models were developed to investigate whether beverage consumption explained variability in smoking and vaping behaviour in adolescence: (1) smoking initiation, (2) vaping initiation, (3) current smoking status, and (4) current vaping status. Models were adjusted for demographic factors. Multinomial logit models were used for model 1, 2, and 3. A binary logistic regression model was used for model 4. An association between change in frequency of beverage consumption and smoking/vaping behaviour was identified in all models. A one-day increase in beverage consumption was associated with smoking initiation (OR = 1.38, 95% CI: 1.25, 1.51), vaping initiation (OR = 1.23, 95% CI: 1.14, 1.32), identifying as a current smoker (OR = 1.17, 95% CI: 1.01, 1.35), and currently vaping (OR = 1.08, 95% CI: 1.04, 1.11). Change in high-energy drink consumption was the best predictor of smoking behaviours and vaping initiation but not current vaping status. Given the health consequences of smoking and vaping and their association with high-energy drink and coffee consumption, policy initiatives to prevent smoking/vaping initiation, and to limit youth access to these beverages, warrant consideration.

## 1. Introduction

Tobacco consumption is the direct cause of ~7 million deaths annually worldwide [1]. More specifically, in Canada, cigarette smoking is the largest modifiable risk factor contributing to the burden of disease (including premature death and disability-adjusted life years) [2]. Approximately 15% of Canadians are considered cigarette smokers, including 10.6% of Canadian youth (aged 15–19) [3]. Smoking prevalence among Canadian youth is especially a concern as early adoption of smoking behaviour decreases the likelihood of quitting smoking in adulthood [4]. Further, many Canadians have vaped, with adolescents and young adults reporting the highest rate of vaping of any age category, with 23% of students in grades 7–12 having tried vaping [5]. Vaping in the youth population is also a risk factor for subsequent cigarette smoking [6,7]. Preventing smoking and vaping initiation during adolescence should be considered a public health priority.

Adolescence is a key transitional phase in development, with increased autonomy potentially impacting behaviour. Increased autonomy results in a wider variety of choices that can lead to experimentation. This may be true for both cigarette smoking and other health behaviours, including beverage consumption, many of which contain caffeine. Caffeine intake begins to increase at ~13 years of age and continues to increase steadily until ~56 years old [8]. The majority (~73%) of US youth consume caffeine on a daily basis [9], primarily in the form of sugar-sweetened beverages (SSBs), high-energy drinks, and coffee [9]. Increased SSB consumption is associated with a range of adverse health outcomes, such as Type II diabetes, cardiovascular disease, and obesity [10]. High-energy drinks have shown acute adverse effects in which consumers report difficulties sleeping, rapid heart rate, chest pain, nausea, and seizures [11].

Conversely, adults’ primary source of caffeine is coffee [12], and the association between coffee consumption and smoking behaviour has been established, with smokers being more likely to consume caffeine than non-smokers [13]. There appears to be a dose–response relationship, with each additional cigarette smoked per day being associated with an additional 0.10 cups of coffee consumed [14]. Both physiological and psychological mechanisms have been proposed to explain why caffeine consumption impacts nicotine use. In animal models, chronic exposure to caffeine potentiates nicotine self-administration [15,16,17], suggesting a cumulative stimulation of dopamine through adenosine receptor antagonization (caffeine) and cholinergic stimulation (nicotine) [15]. Nicotine may also shorten the half-life of caffeine [18], prompting smokers to consume more caffeine. Conditioning may also play a role in this relationship, where coffee consumption triggers a conditioned response to smoke a cigarette [19]. Alternatively, high-energy drink consumption and smoking behaviours have been identified as risky behaviours [20,21]. When one participates in one risky behaviour, their likelihood to participate in others is higher [22]. Mental health symptoms (i.e., stress, anxiety, and depression) have been shown to play a role in both consumption of SSBs and smoking behaviours [23,24,25,26]. Finally, during adolescence, social influences (e.g., peer pressure) have been related to substance use [27]. 

Our research team have established an association between beverage consumption and smoking and vaping behaviour in a cross-sectional analysis of a Canadian sample of high school students [28]. We demonstrated that high-energy drinks have the strongest association with cigarette smoking and vaping out of all of the examined beverages (e.g., coffee/tea with and without sugar and SSBs) [28]. This study identified that the association in this sample was larger, in general, for vaping when compared to smoking. However, this study’s cross-sectional nature did not allow us to determine the potential temporal nature of this relationship. As far as we are aware, one study in Finnish adolescents demonstrated that high-energy drink consumption predicted later smoking and vaping [29]. This study did not examine the association between other types of SSBs or other caffeinated drinks but rather focused only on high-energy drinks. Additionally, smoking rates are higher in Finland compared to Canada (18.2% CI:14.4–21.8 vs. 13.7% CI: 11.5–16.2, respectively) [30]; SSB consumption differs in Europe and North America as well [31]. As such, we will examine the longitudinal association between beverage consumption (i.e., SSB, high-energy drink, and coffee/tea) and smoking/vaping behaviours, with particular attention paid to both high-energy drinks and vaping. We hypothesize that an increased frequency of caffeinated beverages/SSB consumption will be associated with smoking behaviours in youth after controlling for grade, sex, BMI, school clustering, and ethnic background. More specifically, high-energy drink consumption will be the strongest predictor of smoking/vaping behaviours (i.e., smoking initiation, vaping initiation, current/former smokers, current vape users). 

## 2. Methods

### 2.1. Data

This study uses longitudinal-linked, student-level data from the three waves of data collected in the COMPASS system (2015/16 (Time Control (Time C); used to ensure that no smokers or vapers were included in analysis), 2016/17 (Time 1); 2017/18 (Time 2)) to examine if changes from Time 1 to Time 2 in beverage consumption were associated with smoking and vaping at Time 2. The COMPASS study uses self-generated identification codes to anonymously link student data across all years [32]. After the self-generated identification codes are created, they link an individual’s data across two time points. Finally, these data are then coupled together to create a period longer than two years (for example, Time C–Time 1, Time 1–Time 2 prior to Time C–Time 2). All procedures were approved by the University of Waterloo Office of Research Ethics (reference number 30118), appropriate provincial ethics committees, and required school board committees, including passive consent. A full description of the COMPASS study methods is available in print [33] and online (www.compass.uwaterloo.ca; accessed on 7 January 2021).

### 2.2. Data Collection Tools

The student-level questionnaire for COMPASS was used to acquire all data in the current study, besides school median income. The COMPASS questionnaire collects individual student data pertaining to multiple behavioural domains, correlates of the behaviours, and demographic characteristics. In each school, the COMPASS questionnaire was administered during class time to collect within-school samples. The COMPASS questionnaire items are based on national standard or current national public health guidelines [33]. School median income was determined using the first three alphanumeric digits of the postal code of the school and available regional median income data.

### 2.3. Measures

#### 2.3.1. Outcomes

Using the COMPASS questionnaire, two smoking behaviour-related and two vaping behaviour-related outcome variables were identified: smoking initiation, vaping initiation, smoking status, and vaping status. Smoking status at an individual time point was determined through the validated measure [34] for smoking status identification in the COMPASS study. Current smokers were characterized through two questions: “Have you ever smoked 100 or more whole cigarettes in your life?” and “On how many of the last 30 days did you smoke one or more cigarettes?”. Three categories of smoking status were created: current, former, and non-smokers. 

Similarly, current vape users were identified by the questions “Have you ever tried an electronic cigarette, also known as an e-cigarette?” and “On how many of the last 30 days have you used an e-cigarette?” Due to the nature of these questions, 2 vaping usage categories were created: currently vaping and currently not-vaping. At present, only the combustible cigarette smoking status has been validated in the COMPASS data set [34]. 

For longitudinal analysis, Time C was used to remove previous smokers from the analysis. Smoking initiation was identified by individuals who were not smoking at Time 1 but were identified as smokers at Time 2. Vaping initiation was identified in the same manner using the vaping variables.

#### 2.3.2. Beverage Consumption Behaviour

Four beverage consumption behaviours were identified using the available COMPASS questionnaire: frequency of SSB consumption, frequency of high-energy drink consumption, frequency of coffee and tea with sugar consumption, frequency of coffee and tea without sugar consumption. This is similar to the preceding cross-sectional study on this topic [28]. For detailed descriptions of the questions, please refer to the study by Fagan and colleagues [28]. In addition to the 5 weekdays of beverage consumption (Monday–Friday) used by Fagan et al. [28], we included weekend beverage consumption as well (Saturday–Sunday). Weekday and weekend day are combined in the current analysis. Response options included: none, 1 day, 2 days. Therefore, the final summed variable ranged from none (0 days) to 7 days.

### 2.4. Covariates

Demographic characteristics, including grade (9, 10, 11, 12), ethnicity (white, black, Indigenous, Asian, Hispanic/Latin), sex (female, male), school area median income, and body mass index (BMI) calculated from self-reported height (cm) and weight (kg), were controlled for in all models of the analysis. 

### 2.5. Statistical Analysis 

Descriptive statistics were used to assess differences in demographics and beverage consumption between students who engaged in smoking behaviours and students who did not (Student’s t-test for continuous variables and chi-square test for categorical variables) for each model. If variables were skewed, the Wilcoxon rank sum test was used. All analyses were run using SAS software package 9.4 (Cary, NC, USA).

Four separate models were developed for (1) smoking initiation, (2) vaping initiation, (3) current/former smokers, and (4) current vape users. Multinomial logit models were used for model 1, 2, and 3 and a binary logistic regression model was used for Model 4. These models focused on the change in beverage consumption from Time 1 (2016–2017) to Time 2 (2017–2018). This allows the model to account for beverage consumption at both time points to predict subsequent smoking/vaping behaviours at Time 2 (2017–2018). Change in beverage consumption was used, rather than a level of beverage consumption, in an attempt to control for discrepancies in the starting points of students’ beverage consumption at Time 1 and to uncover the longitudinal effects (2016–2017 to 2017–2018). All models included a cross-sectional analysis that included the level of beverage consumption at Time 1 (please see Supplementary Tables S1–S4). The level of beverage consumption at Time 1 was controlled for in the longitudinal analysis. Model 1 and 2 explicitly control for smoking/vaping at Time 1 (as we are looking for smoking and vaping initiation), whereas Model 3 implicitly controls for smoking at Time 1, as the COMPASS classifications identify current smokers, former smokers, and never smokers, thus implicitly accounting for prior smoking behaviours. Model 4 implicitly controls for vaping at Time 1, as two classifications are created: non-vapers and vapers. To be classified as a non-vaper, a participant must have self-reported never vaping in their lifetime. Only non-smokers and non-vapers at Time C (2015/16) were included in the analysis. PROC GENMOD in SAS was used to analyze each model. School clustering was included in the model with independent working correlation. Test results based on empirical variance estimates were presented. The significance level was set at 0.05 (two-sided test). Each beverage type was tested individually in the models. All beverage variables were adjusted for their means to reflect the longitudinal effects. In smoking models, the non-smoker group was the referent. In vaping models, the non-vape user group was the referent. 

## 3. Results

### 3.1. Demographic and Descriptive Statistics

At Time 1, the majority of participants identified as white (females 73.1%, males 71.7%), and as living in a large urban location (females 51.8% and 52.6% males). Demographic characteristics at Time 1 for the linked longitudinal sample are shown in Table 1. Descriptive statistics indicate that the prevalence of current vaping at Time 2 (11.4%) was higher than the prevalence of current cigarette use (6.3%). Additional descriptive/demographic statistics for each model are presented in Table 2 (Model 1), Table 3 (Model 2), and Table 4 (Model 3 and 4) as demographic characteristics vary slightly in each model due to different patterns of missing data. For descriptive statistics on beverage consumption and smoking/vaping behaviours, please see Tables S5 and S6 in the supplementary file.

### 3.2. Modelling Analysis

Based on the longitudinal effects of the models, it was found that a one-day increase in beverage consumption (including high-energy drinks, coffee and tea with/without sugar) was associated with increased odds of smoking and vaping behaviour one year later. The type of beverage most strongly related to smoking and vaping behaviour for this longitudinal association was high-energy drinks, followed by coffee and tea with sugar, coffee and tea without sugar, and SSB consumption. For the covariate effects for each model, please see Tables S1–S4 in the supplementary file. 

#### 3.2.1. Model 1

Model 1 assessed the longitudinal association between change in beverage consumption (Time 1 and Time 2) and smoking initiation between Time 1 and Time 2. A one-day increase in consuming energy drinks provided the largest OR for smoking initiation (OR = 1.38, 95% CI: 1.25, 1.51), followed by coffee and tea with sugar consumption (OR = 1.10, 95% CI: 1.06, 1.14), and coffee and tea without sugar consumption (OR = 1.07, 95% CI: 1.02, 1.12). SSB consumption was not significantly associated with smoking initiation. For full Model 1 results, please see Table 5. 

#### 3.2.2. Model 2

Model 2 assessed the longitudinal association between change in beverage consumption (Time 1–Time 2) and vaping initiation from Time 1 to Time 2. Similar to what was seen in Model 1, a one-day increase in high-energy drink consumption demonstrated the greatest increase in the odds of initiating vaping (OR = 1.23, 95% CI: 1.14, 1.32), followed by coffee and tea with sugar consumption (OR = 1.08, 95% CI: 1.05, 1.12). Coffee and tea without sugar and SSB consumption were not significantly associated with smoking initiation. For full Model 2 results, please see Table 6. 

#### 3.2.3. Model 3

Model 3 assessed the longitudinal association between change in beverage consumption (Time 1–Time 2) and current smoking status at Time 2. A one-day increase in high-energy drink consumption increased the odds of being a current smoker (OR = 1.17, 95% CI: 1.01, 1.35). None of the other changes in beverage consumption were significantly associated with current smoking. For full Model 3 results, please see Table 7. 

#### 3.2.4. Model 4

Model 4 assessed the longitudinal association between change in beverage consumption (Time 1–Time 2) and current vaping status at Time 2. Unlike Model 3, a one-day increase in coffee and tea with sugar consumption provided the largest OR for being a current vape user (OR = 1.08, 95% CI: 1.04, 1.11), followed by coffee and tea without sugar consumption (OR = 1.07, 95% CI: 1.02, 1.11) and high-energy drink consumption (OR = 1.07, 95% CI: 1.00, 1.14). SSB consumption was not significantly associated with vaping status. For full Model 4 results, please see Table 8. 

## 4. Discussion

Our study provides evidence for the longitudinal associations of increased caffeinated beverage consumption and smoking and vaping behaviours in a large prospective cohort study of Canadian youth. In line with our hypothesis, a one-day increase in high-energy drink consumption was associated with cigarette and vaping initiation, and with the status of cigarette smoking and vaping use, after controlling for grade, sex, BMI, school median income, and ethnicity. Similar results were seen for coffee and tea consumption with or without sugar. However, no significant association was identified with current smoking status or vaping use and coffee and tea without sugar consumption. Contrary to our hypothesis, little evidence was found for the longitudinal effects of SSB on smoking or vaping behaviours. 

With the significant health burden that smoking cigarettes creates in Canada [2], and the recent surge in vaping, where the adverse health risks are slowly being realized [35], it is vital to uncover what may be preceding this behaviour in youth. We hypothesized that beverage consumption and, in particular, caffeinated beverage consumption might enhance smoking and vaping behaviour in youth as beverage consumption, especially beverages that contain caffeine, has been associated with smoking behaviours in adults [13,36]. Additionally, adult smokers consume more sugar than a non-smoker, which may be caused by an altered taste due to smoking, and the presence of sugar in tobacco products can facilitate smoking behaviour [37,38,39]. Adolescence and young adulthood are associated with increasing autonomy over food and beverage consumption and with viewing more advertisements for beverages containing caffeine [40]. This period is associated with increased caffeine and SSB consumption [41,42,43]. With the many beverages available to youth, it is important to identify which beverages may impact smoking and vaping behaviour. 

High-energy drinks have been associated with drug use and smoking in young adults [44,45] and have been longitudinally associated with smoking and vaping in a sample of Finnish adolescence [29]. This longitudinal study (n = 5742) found that daily energy drink use was associated with ever vaping more than twice (multivariate model OR = 2.36 95% CI: 1.50–3.70 for boys; multivariate model OR = 3.94, 95% CI: 1.66–9.32 for girls) and smoking more than 50 cigarettes (multivariate model OR = 1.80, 95% CI: 1.06–3.05 for boys; multivariate model OR = 1.87, 95% CI: 0.75–4.63 for girls). Our study builds on these findings by showing that other beverages that contain caffeine are longitudinally associated with smoking and vaping behaviours, suggesting that caffeine, and not sugar, may be influential in these associations. 

Interestingly, our study found little evidence for change in SSB being a risk factor for subsequent smoking and vaping behaviours. This is not consistent with the preceding cross-sectional study [28], which saw an association between SSB and smoking and vaping behaviours. Other cross-sectional work has shown a link between SSB and smoking behaviours in adults. In a large American cohort (n = 12,214), nicotine consumption was associated with increased SSB consumption in a young adult population (mean age: ~24 years) [46]. This was also seen in a general adult population as SSB consumption predicted smoking behaviour [47]. Potential reasons for this may be the broad classification of SSBs in the COMPASS study, which includes flavoured milk, for example. Not all of the SSBs in this category contain caffeine, as the question prompts students with examples of soda pop, Gatorade, and Kool-Aid (2/3 do not contain caffeine). If it is caffeine and not sugar that is driving the association between beverage consumption and smoking and vaping behaviour, the large number of SSBs not containing caffeine examined in the COMPASS questionnaire may weaken potential associations in the current study. The observed association between high-energy drink consumption, a subtype of SSB and a large source of caffeine in youth [45], and smoking and vaping behaviour supports this hypothesis. 

The physiological [13,14,15,16,17,18,19] and psychological [20,21,23,24,25,26,27] mechanisms documented in the literature also support this hypothesis. As mentioned above, chronic exposure to caffeine potentiates nicotine self-administration [15,16,17], suggesting a cumulative stimulation of the dopamine through adenosine receptor antagonization (caffeine) and cholinergic stimulation (nicotine) [15]. High-energy drink consumption and smoking/vaping behaviours have been identified as risky behaviours [20,21], and youth who participate in one risky behaviour are more likely to participate in other risky behaviours. Stress, anxiety, and depression have been linked to both SSBs and smoking behaviours [23,24,25,26] and peer pressure has also been related to substance use in adolescence [27]. 

The findings have implications for practice and policy and the need to consider the inter-relationships between smoking behaviour, particularly vaping use, and caffeinated beverage consumption. There has been much progress in preventing cigarette use in Canada, such as sales restriction to anyone under the age of 18, graphic warning labels taking up 75% of the packaging space, plain packaging, and the prohibition of most forms of advertising [48]. Vaping products are subject to many of the same restrictions, with one notable exception: graphic warning labels are not used on vaping packaging and simple, text-only warnings are required [48]. The lack of graphic warning labels may contribute to many students being more willing to try the product. However, it is the aggressive marketing of vaping that is suspected to have driven the recent increase in their use. The vaping market was liberalized in Canada in 2018. Between 2017 and 2019, the percentage of youth who reported noticing vaping promotions often or very often doubled from 13.6% to 26.0%. Youth who reported noticing marketing often or very often were more likely to report vaping in the past 30 days, past week, and on >20 days in the last month [49]. In Canada, provinces that have lower restrictions on the marketing of vaping products saw higher percentages of vaping [49]. This suggests an association between the exposure to vaping marketing and its use among youth. Greater regulation of marketing is required to reduce vaping use in adolescents. 

Marketing may also play a role in the consumption of high-energy drinks. For example, the primary target of energy drink marketing is teenagers and young adults aged 18–34 years old [50]. This age group generally lead busy lifestyles and are receptive to the types of advertisements that these products usually employ. Energy drinks are exceptionally popular among young adults as 34% of 18- to 24-year-olds are considered regular users [51]. The marketing for these brands reflects their demographic, using cross-promotional techniques by integrating their products with things that appeal to young adults, such as extreme sports and popular culture icons [51]. Youth are not immune to these marketing efforts. In a study evaluating the exposure of Canadian youth and young adults (age: 12–24) to high-energy drink marketing, over 80% of respondents reported ever seeing energy drink marketing; conversely, only 32% of survey respondents reported ever seeing education massages about the potential harms of energy drinks [52]. Increased exposure to energy drink marketing is associated with increased consumption in adults [53]. Enforcing responsible marketing and increasing education surrounding the risks of consuming energy drinks and vaping use will be necessary tools as part of a comprehensive strategy to reduce the use of both these substances.

### Limitations

This is the first study to examine the longitudinal effects of a one-day increase in beverage consumption on smoking and vaping behaviours in a large national sample of Canadian youth. However, some limitations should be acknowledged. A ceiling effect, where students with high frequency of beverage consumption at Time 1 are less likely to demonstrate a large increase in beverage consumption, may explain why no associations were identified for SSBs. To account for this, we reported the ORs for a one-day increase in beverage consumption. The COMPASS study uses self-report measures, which can be subject to error. Additionally, social desirability bias may have impacted how the students self-reported beverage and vaping consumption. Academic achievement was not used as a covariate due to the lack of variability in the self-reported response. The beverage consumption measures were not validated, and the sugar-sweetened beverages question did not differentiate between caffeinated and non-caffeinated drinks. 

## 5. Conclusions

With the health consequences associated with smoking/vaping behaviours and the recent uptake in vaping use in youth, it is essential to try and find ways to mitigate these behaviours. This study shows a prospective association between a one-day increase in caffeinated beverage consumption and smoking/vaping initiation. Stronger policy consideration is particularly required regarding the marketing towards youth of vaping products and high-energy drinks. Interventions may also be useful in the school setting that educate young adults as to the potential harmful consequences of their consumption. 

## Figures and Tables

**Table 1:** Descriptive statistics at Time 1 (2016/2017) for the longitudinal sample of students in the COMPASS study in our analysis.

		Females	Males
GRADE		N(%)	N(%)
	9	13(0.2)	14(0.3)
	10	1694(27.2)	1510(28.9)
	11	3025(49.4)	2573(48.9)
	12	1386(22.7)	1166(22.2)
LOCATION		N(%)	N(%)
	Large urban	3172(51.8)	2768(52.6)
	Medium urban	1211(19.8)	1006(19.1)
	Small/Rural	1736(28.4)	1489(28.3)
RACE		N(%)	N(%)
	White	4473(73.1)	3773(71.7)
	Black	206(3.4)	200(3.8)
	Asian	360(5.9)	419(8.0)
	Hispanic	146(5.4)	137(2.6)
	Other/mixed	934(15.3)	734(13.9)
SCHOOL MEDIAN INCOME ($)		N(%)	N(%)
	25,001–50,000	753(12.3)	622(11.8)
	50,001–75,000	2844(46.5)	2442(46.4)
	75,001–100,000	2042(33.4)	1772(33.7)
	>100,000	480(7.8)	427(8.1)

Note. BMI = body mass index. SD = standard deviation.

**Table 2:** Demographic and descriptive statistics of participants at Time 1 (2016/17) for smoking initiation in the COMPASS study at Time 2 (2017/18).

		Females	Males
GRADE		N(%)	N(%)
	9	2(0.1)	4(0.2)
	10	74(2.8)	66(2.9)
	11	1487(55.8)	1262(55.8)
	12	1101(41.3)	929(41.1)
LOCATION		N(%)	N(%)
	Large urban	1426(53.5)	1219(53.9)
	Medium urban	526(19.7)	428(18.9)
	Small/Rural	712(26.7)	614(27.2)
BMI		N(%)	N(%)
	Underweight	34(1.3)	39(1.7)
	Healthy weight	1645(61.7)	1346(59.5)
	Overweight	380(1.3)	307(13.6)
	Obese	157(5.9)	178(7.9)
	Not stated	448(16.8)	391(17.3)
RACE N(%)		N(%)	N(%)
	White	1973(74.1)	1625(71.9)
	Black	87(3.3)	77(3.4)
	Asian	167(6.3)	186(8.2)
	Hispanic	68(2.6)	61(2.7)
	Other/mixed	369(13.9)	312(13.8)
SCHOOL MEDIAN INCOME ($)		N(%)	N(%)
	25,001–50,000	352(13.2)	284(12.6)
	50,001–75,000	1230(46.2)	1032(45.6)
	75,001–100,000	876(32.9)	762(33.7)
	>100,000	206(7.7)	183(8.1)
DAYS PER SCHOOL WEEK DRINKING SSB		M(SD)	M(SD)
		2.15(2.16)	2.26(2.16)
DAYS PER SCHOOL WEEK DRINKING HIGH-ENERGY DRINKS		M(SD)	M(SD)
		0.26(0.96)	0.32(1.05)
DAYS PER SCHOOL WEEK DRINKING COFFEE/TEA WITH SUGAR		M(SD)	M(SD)
		2.06(2.38)	1.93(2.35)
DAYS PER SCHOOL WEEK DRINKING COFFEE/TEA WITHOUT SUGAR		M(SD)	M(SD)
		1.05(2.08)	0.85(1.88)
DIFFERENCE DAY PER WEEK DRINKING SSB		M(SD)	M(SD)
		−0.36(2.96)	0.34(3.09)
DIFFERENCE DAYS PER WEEK DRINKING HIGH-ENERGY DRINKS		M(SD)	M(SD)
		−0.12(3.30)	0.03(1.47)
DIFFERENCE DAYS PER WEEK DRINKING COFFEE/TEA WITH SUGAR		M(SD)	M(SD)
		0.12(3.30)	−0.35(3.21)
DIFFERENCE DAY PER WEEK DRINKING COFFEE/TEA WITHOUT SUGAR		M(SD)	M(SD)
		0.06(2.96)	−0.17(2.54)
SMOKING CLASSIFICATION		N(%)	N(%)
	NO–NO	2169(81.4)	1735(76.7)
	NO–YES	33(1.2)	35(1.5)
	YES–NO	265(9.9)	276(12.2)
	YES–YES	197(7.4)	215(9.5)

Note. BMI = body mass index. SD = standard deviation.

**Table 3:** Demographic and descriptive statistics of participants at Time 1 (2016/17) for vaping initiation in the COMPASS study at Time 2 (2017/18).

		Females	Males
GRADE		N(%)	N(%)
	9	2(0.1)	4(0.2)
	10	83(2.9)	67(2.8)
	11	1591(55.4)	1319(55.3)
	12	1195(41.6)	994(41.7)
LOCATION		N(%)	N(%)
	Large urban	1492(52.0)	1272(53.4)
	Medium urban	574(20.0)	454(19.0)
	Small/Rural	805(28.0)	658(27.6)
BMI		N(%)	N(%)
	Underweight	35(1.2)	41(1.7)
	Healthy weight	1751(61.0)	1414(59.3)
	Overweight	416(14.5)	315(13.2)
	Obese	178(6.2)	190(8.0)
	Not stated	491(17.1)	424(17.8)
RACE N(%)		N(%)	N(%)
	White	2112(73.6)	1701(71.4)
	Black	93(3.2)	84(3.5)
	Asian	172(6.0)	191(8.0)
	Hispanic	70(2.4)	65(2.7)
	Other/mixed	424(14.8)	343(14.4)
SCHOOL MEDIAN INCOME ($)		N(%)	N(%)
	25,001–50,000	361(12.6)	292(12.2)
	50,001–75,000	1333(46.4)	1087(45.6)
	75,001–100,000	957(33.3)	814(34.1)
	>100,000	220(7.7)	191(8.0)
DAYS PER SCHOOL WEEK DRINKING SSB		M(SD)	M(SD)
		2.16(2.17)	2.29(2.17)
DAYS PER SCHOOL WEEK DRINKING HIGH-ENERGY DRINKS		M(SD)	M(SD)
		0.26(0.96)	0.35(1.12)
DAYS PER SCHOOL WEEK DRINKING COFFEE/TEA WITH SUGAR		M(SD)	M(SD)
		2.06(2.39)	1.95(2.35)
DAYS PER SCHOOL WEEK DRINKING COFFEE/TEA WITHOUT SUGAR		M(SD)	M(SD)
		1.05(2.07)	0.84(1.87)
DIFFERENCE DAY PER WEEK DRINKING SSB		M(SD)	M(SD)
		−0.34(2.97)	0.31(3.10)
DIFFERENCE DAYS PER WEEK DRINKING HIGH-ENERGY DRINKS		M(SD)	M(SD)
		−0.10(1.19)	0.03(1.53)
DIFFERENCE DAYS PER WEEK DRINKING COFFEE/TEA WITH SUGAR		M(SD)	M(SD)
		0.16(3.32)	−0.34(3.24)
DIFFERENCE DAY PER WEEK DRINKING COFFEE/TEA WITHOUT SUGAR		M(SD)	M(SD)
		0.07(2.93)	−0.13(2.54)
VAPING CLASSIFICATION		N(%)	N(%)
	NO–NO	2421(84.3)	1820(76.3)
	NO–YES	59(2.1)	51(2.1)
	YES–NO	319(11.1)	378(15.9)
	YES–YES	72(2.5)	315(5.7)

Note. M = mean. SD = standard deviation. N = number of participants. % = percent of the group. SSB = sugar sweetened beverages.

**Table 4:** Demographic and descriptive statistics of participants at Time 1 (2016/17) for current smoking and vaping status in the COMPASS study at Time 2 (2017/18).

		Females	Males
GRADE		N(%)	N(%)
	9	1726(52.5)	1562(53.9)
	10	1436(43.7)	1186(40.9)
	11	120(3.6)	140(4.8)
	12	6(0.2)	12(0.4)
LOCATION		N(%)	N(%)
	Large urban	1704(51.8)	1514(52.2)
	Medium urban	644(19.6)	553(19.1)
	Small/Rural	941(28.6)	833(28.7)
BMI		N(%)	N(%)
	Underweight	39(1.2)	51(1.8)
	Normal weight	1993(60.5)	1711(59.1)
	Overweight	476(14.5)	392(13.5)
	Obese	205(6.2)	234(8.1)
	Not stated	579(17.6)	508(17.5)
RACE		N(%)	N(%)
	White	2391(72.7)	2089(72.0)
	Black	120(3.6)	109(3.5)
	Asian	184(5.6)	220(7.6)
	Hispanic	79(2.4)	73(2.5)
	Other/mixed	515(15.7)	409(14.1)
SCHOOL MEDIAN INCOME ($)		N(%)	N(%)
	25,001–50,000	400(12.2)	339(11.7)
	50,001–75,000	1521(46.2)	1352(46.6)
	75,001–100,000	1105(33.6)	977(33.7)
	>100,000	263(8.0)	232(8.0)
DAYS PER SCHOOL WEEK DRINKING SSB		M(SD)	M(SD)
		2.14(2.02)	2.80(2.15)
DAYS PER SCHOOL WEEK DRINKING HIGH-ENERGY DRINKS		M(SD)	M(SD)
		0.12(0.57)	0.29(0.98)
DAYS PER SCHOOL WEEK DRINKING COFFEE/TEA WITH SUGAR		M(SD)	M(SD)
		1.87(2.16)	1.29(2.04)
DAYS PER SCHOOL WEEK DRINKING COFFEE/TEA WITHOUT SUGAR		M(SD)	M(SD)
		0.91(1.88)	0.44(1.34)
SMOKING DAYS IN LAST MONTH		N(%)	N(%)
	1 (0 days)	3163(96.7)	2783(96.5)
	2 (1 day)	64(2.0)	46(1.6)
	3 (2–3 days)	27(0.8)	31(1.1)
	4 (4–5 days)	5(0.2)	10(0.3)
	5 (6–10 days)	6(0.2)	5(0.2)
	6 (11–20 days)	3(0.1)	1(0.0)
	7 (21 to 29 days)	1(0.0)	2(0.1)
	8 (everyday)	2(0.1)	7(0.2)
VAPING DAYS IN LAST MONTH		N(%)	N(%)
	1 (0 days)	3127(96.9)	2633(92.6)
	2 (1 day)	52(1.6)	105(3.7)
	3 (2–3 days)	27(0.8)	40(1.4)
	4 (4–5 days)	10(0.3)	19(0.7)
	5 (6–10 days)	7(0.2)	25(0.9)
	6 (11–20 days)	2(0.1)	9(0.3)
	7 (21 to 29 days)	1(0.0)	5(0.2)
	8 (everyday)	2(0.1)	6(0.2)

Note. M = mean. SD = standard deviation. N = number of participants. % = percent of group. SSB = sugar sweetened beverages.

**Table 5:** Model 1: Longitudinal association between change in beverage consumption (2016/17–2017/18) and smoking initiation (2016/17–2017/18) in the COMPASS study.

Parameter	Smoking Group	*p*-Value	OR (95% CI)
Change in SSB consumption	No–no	(REF)	-
	No–yes	0.3444	0.98(0.93, 1.03)
	Yes–no	0.4197	0.94(0.82, 1.08)
	Yes–yes	0.1358	1.04(0.99, 1.08)
Change in high-energy drink consumption	No–no	(REF)	-
	No–yes	<0.001	1.37(1.25, 1.51)
	Yes–no	0.7310	1.04(0.82, 1.32)
	Yes–yes	<0.001	1.37(1.25, 1.49)
Change in coffee and tea with sugar	No–no	(REF)	-
	No–yes	<0.001	1.10(1.05, 1.14)
	Yes–no	0.00059	1.19(1.08, 1.31)
	Yes–yes	<0.001	1.12(1.08, 1.16)
Change in coffee and tea without sugar	No–no	(REF)	-
	No–yes	0.00759	1.07(1.02, 1.12)
	Yes–no	0.9353	1.00(0.90, 1.12)
	Yes–yes	0.06140	1.05(1.00, 1.11)

Note: OR = Odds ratio. 95% CI = 95 percent confidence interval. REF = reference group for the model.

**Table 6:** Model 2: Longitudinal association between change in beverage consumption (2016/17–2017/18) and vaping initiation (2016/17–2017/18) in the COMPASS study.

Parameter	E-Cig Group	*p*-Value	OR (95% CI)
Change in SSB consumption	No–no	(REF)	-
	No–yes	0.9710	1.00(0.96, 1.04)
	Yes–no	0.12919	1.06(0.98, 1.14)
	Yes–yes	0.4786	1.02(0.96, 1.09)
Change in high-energy drink consumption	No–no	(REF)	-
	No–yes	<0.001	1.23(1.14, 1.33)
	Yes–no	<0.001	1.26(1.13, 1.40)
	Yes–yes	<0.001	1.34(1.22, 1.48)
Change in coffee and tea with sugar	No–no	(REF)	-
	No–yes	<0.001	1.08(1.05, 1.12)
	Yes–no	0.291134	1.05(0.96, 1.14)
	Yes–yes	<0.001	1.15(1.10, 1.21)
Change in coffee and tea without sugar	No–no	(REF)	-
	No–yes	0.25404	1.03(0.98, 1.08)
	Yes–no	0.02165	1.10(1.01, 1.19)
	Yes–yes	0.37453	1.04(0.96, 1.13)

Note: OR = Odds ratio. 95% CI = 95 percent confidence interval. REF = reference group for the model.

**Table 7:** Model 3: Longitudinal association between change in beverage consumption (2016/17–2017/18) and never smokers, current smokers, and former smokers in 2017/2018 in the COMPASS study.

Parameter	Smoking Group	*p*-Value	OR (95% CI)
Change in SSB consumption	Never smoker	(REF)	-
	Current smoker	0.9431	1.01(0.87, 1.17)
	Former smoker	0.6470	1.13(0.67, 1.91)
Change in high-energy drink consumption	Never smoker	(REF)	-
	Current smoker	0.22976	1.13(0.92, 1.38)
	Former smoker	0.0206	0.58(0.36,0.92)
Change in coffee and tea with sugar	Never smoker	(REF)	-
	Current smoker	0.23162	0.92(0.81, 1.05)
	Former smoker	0.2723	0.88(0.70, 1.11)
Change in coffee and tea without sugar	Never smoker	(REF)	-
	Current smoker	0.2639	0.91(0.77, 1.07)
	Former smoker	0.7852	1.05(0.72, 1.54)

Note: OR = Odds ratio. 95% CI = 95 percent confidence interval. REF = reference group for the model.

**Table 8:** Model 4: Longitudinal association between change in beverage consumption (2016/17–2017/18) and current vaping status (2017/2018) in the COMPASS study.

Parameter	E-Cig group	*p*-Value	OR (95% CI)
Change in SSB consumption	No	(REF)	-
	Yes	0.1276	0.96(0.92, 1.01)
Change in high-energy drink consumption	No	(REF)	-
	Yes	0.12075	1.06(0.98, 1.15)
Change in coffee and tea with sugar	No	(REF)	-
	Yes	<0.001	1.07(1.03, 1.12)
Change in coffee and tea without sugar	No	(REF)	-
	Yes	<0.001	1.08(1.02, 1.14)

Note: OR = Odds ratio. 95% CI = 95 percent confidence interval. REF = reference group for the model.

## Data Availability

The datasets generated and analyzed for this study will not currently be shared because this is an ongoing study; however, access to the data supporting the findings of this study can be requested at https://uwaterloo.ca/compass-system/information-researchers (accessed on 6 April 2021).

## Supplementary Materials

The following are available online at https://www.mdpi.com/article/10.3390/ijerph18083864/s1, Table S1: Model 1: Longitudinal association between change in beverage consumption (2016/17-2017/18) and smoking initiation (2016/17-2017/18) in the COMPASS study with covariate effects; Table S2: Model 2: Longitudinal association between change in beverage consumption (2016/17-2017/18) and vaping initiation (2016/17-2017/18) in the COMPASS study covariate effects; Table S3: Model 3: Longitudinal association and covariate effects between change in beverage consumption (2016/17-2017/2018) and never smokers, current smokers and former smokers at 2017/2018 in the COMPASS study; Table S4: Model 4: Longitudinal association and covariate effects between change in beverage consumption (2016/17-2017/2018) and current vaping status at Time 3 (2017/2018) in the COMPASS study; Table S5: Descriptive statistics of beverage consumption at all times and smoking at all time points for linked data in the study; Table S6: Frequency and percent of beverage consumption at all times and smoking at all time points for entire linked sample in the study.
