# Supplementary material for: The Energy to Smoke: Examining the Longitudinal Association between Beverage Consumption and Smoking and Vaping Behaviours among Youth in the COMPASS Study
Source: Int J Environ Res Public Health. 2021 Apr 7;18(8):3864. doi: 10.3390/ijerph18083864 (PMC8067730; doi:10.3390/ijerph18083864)
Supplement: Supplementary file 1 [file ijerph-18-03864-s001.pdf]

Supplementary

Table S1. Model 1: Longitudinal association between change in beverage consumption (2016/17-2017/18) and smoking initiation (2016/17-2017/18) in the COMPASS study with covariate effects.

		**Initiated Smoking (No-Yes)**		**Quit Smoking (Yes-No)**		**Always Smoked (Yes-Yes)**	
Category	Level	OR (CI)	*p*	OR (CI)	*p*	OR (CI)	*p*
Gender	Male	1.36(1.14, 1.63)	<0.001	1.50(0.93, 2.41)	0.093	1.34(1.12, 1.60)	0.001
	Female (REF)	1.00(1.00, 1.00)		1.00(1.00, 1.00)		1.00(1.00, 1.00)	
Grade	10	0.21(0.04, 1.20)	0.078	1357.94(133.89,13772.25)	<0.001	0.47(0.06, 3.36)	0.449
	11	0.19(0.03, 1.03)	0.054	2161.00(654.67, 7133.21)	<0.001	0.32(0.05, 2.09)	0.232
	12	0.20(0.04, 1.09)	0.062	3138.80(937.82,10505.23)	<0.001	0.33(0.05, 2.13)	0.242
	9 (REF)	1.00(1.00, 1.00)		1.00(1.00, 1.00)		1.00(1.00, 1.00)	
Race	Black	0.83(0.46, 1.48)	0.529	2.36(0.92, 6.10)	0.076	0.78(0.37, 1.68)	0.531
	Asian	0.37(0.23, 0.60)	<0.001	1.45(0.61, 3.41)	0.399	0.67(0.38, 1.18)	0.167
	Hispanic	1.31(0.73, 2.34)	0.362	1.44(0.36, 5.68)	0.605	1.02(0.41, 2.54)	0.961
	Other/Mixed/Missing	0.83(0.60, 1.15)	0.270	2.16(1.21, 3.86)	0.010	1.00(0.73, 1.38)	0.982
	White (REF)	1.00(1.00, 1.00)		1.00(1.00, 1.00)		1.00(1.00, 1.00)	
School Median Income	50001-75000	1.87(1.54, 2.27)	<0.001	0.81(0.36, 1.84)	0.620	5.24(3.23, 8.47)	<0.001
	75000-100000	1.87(1.50, 2.34)	<0.001	1.14(0.55, 2.39)	0.722	4.17(2.50, 6.94)	<0.001
	>100000	1.84(0.92, 3.65)	0.082	0.53(0.16, 1.70)	0.284	3.32(1.69, 6.51)	<0.001
	25001-50000	1.00(1.00, 1.00)		1.00(1.00, 1.00)		1.00(1.00, 1.00)	
Location	Large Urban	0.56(0.45, 0.69)	<0.001	0.61(0.34, 1.11)	0.107	0.44(0.29, 0.66)	<0.001
	Medium Urban	1.04(0.69, 1.55)	0.857	0.45(0.22, 0.92)	0.029	0.69(0.38, 1.26)	0.226
	Small/Rural (REF)	1.00(1.00, 1.00)		1.00(1.00, 1.00)		1.00(1.00, 1.00)	
BMI	Not Stated	1.19(0.90, 1.57)	0.229	1.48(0.78, 2.79)	0.228	0.94(0.67, 1.32)	0.729
	Obese	1.13(0.76, 1.70)	0.542	1.80(0.82, 3.98)	0.145	1.07(0.72, 1.57)	0.748
	Overweight	1.00(0.70, 1.44)	0.981	0.91(0.49, 1.69)	0.759	0.91(0.61, 1.36)	0.646
	Underweight	0.49(0.19, 1.29)	0.150	0.96(0.13, 7.23)	0.966	0.29(0.07, 1.23)	0.095
	Healthy Weight (REF)	1.00(1.00, 1.00)		1.00(1.00, 1.00)		1.00(1.00, 1.00)	
Mean SSB		1.01(0.95, 1.06)	0.840	0.97(0.83, 1.14)	0.730	1.08(1.01, 1.15)	0.019
Mean HED		1.38(1.22, 1.57)	<0.001	1.00(0.73, 1.36)	0.985	1.44(1.29, 1.62)	<0.001
Mean CTWS		1.06(1.00, 1.12)	0.032	1.17(1.02, 1.33)	0.020	1.15(1.09, 1.21)	<0.001
Mean CTOS		1.05(0.99, 1.11)	0.121	1.00(0.83, 1.22)	0.965	1.00(0.93, 1.09)	0.917
Difference SSB		0.98(0.93, 1.03)	0.344	0.94(0.82, 1.08)	0.420	1.04(0.99, 1.08)	0.136
Difference HED		1.37(1.25, 1.51)	<0.001	1.04(0.82, 1.32)	0.731	1.37(1.25, 1.49)	<0.001
Difference CTWS		1.10(1.05, 1.14)	<0.001	1.19(1.08, 1.31)	<0.001	1.12(1.08, 1.16)	<0.001
Difference CTOS		1.07(1.02, 1.12)	0.008	1.00(0.90, 1.12)	0.935	1.05(1.00, 1.11)	0.0614

Note. SSB= Sugar-sweetened beverages. CTWS = Coffee and tea with sugar. CTOS = Coffee and tea without sugar. HED= High energy drinks. OR= Odds ratio. CI = 95% Confidence Intervals. BMI= Body mass index. No-Yes= not smoking at time 1 but smoking at time 2. Yes-no = smoking at time 1 and not smoking at time 2. Yes-Yes = smoking at both time 1 and time 2. No-no = not smoking at either time point and is referent. REF = referent.

Table S2. Model 2: Longitudinal association between change in beverage consumption (2016/17-2017/18) and vaping initiation (2016/17-2017/18) in the COMPASS study covariate effects

		**Initiated Vaping (No-Yes)**		**Quit Vaping (Yes-No)**		**Always Vaped (Yes-Yes)**	
Category	Level	OR (CI)	*p*	OR (CI)	*p*	OR (CI)	*p*
Gender	Male	1.63(1.37, 1.94)	<0.001	1.13(0.83, 1.55)	0.440	2.64(1.87, 3.71)	<0.001
	Female (REF)	1.00(1.00, 1.00)		1.00(1.00, 1.00)		1.00(1.00, 1.00)	
Grade	10	1.13(0.12, 10.46)	0.911	7032.67(1449.61, 34118.55)	<0.001	5796.94(873.76,38459.64)	<0.001
	11	0.75(0.09, 6.36)	0.792	10544.06(3954.85, 28111.64)	<0.001	19243.49(5064.54,73118.58)	<0.001
	12	0.89(0.10, 7.63)	0.915	11706.52(4073.48, 33642.64)	<0.001	13814.87(3483.10,54793.33)	<0.001
	9 (REF)	1.00(1.00, 1.00)		1.00(1.00, 1.00)		1.00(1.00, 1.00)	
Race	Black	0.94(0.53, 1.66)	0.828	1.28(0.46, 3.56)	0.631	0.57(0.24, 1.39)	0.219
	Asian	0.62(0.45, 0.85)	0.003	0.86(0.40, 1.84)	0.694	0.31(0.13, 0.73)	0.007
	Hispanic	0.80(0.42, 1.50)	0.487	1.18(0.40, 3.53)	0.763	0.33(0.09, 1.32)	0.117
	Other/Mixed/Missing	0.81(0.64, 1.03)	0.083	1.29(0.75, 2.22)	0.349	0.70(0.47, 1.04)	0.078
	White (REF)	1.00(1.00, 1.00)		1.00(1.00, 1.00)		1.00(1.00, 1.00)	
School Median Income	50001-75000	1.16(0.81, 1.66)	0.420	1.84(1.07, 3.15)	0.026	1.99(0.56, 7.13)	0.288
	75000-100000	0.96(0.71, 1.29)	0.791	1.67(0.97, 2.86)	0.063	1.77(0.54, 5.78)	0.342
	>100000	0.94(0.56, 1.58)	0.825	1.15(0.59, 2.24)	0.687	1.21(0.29, 5.00)	0.793
	25001-50000	1.00(1.00, 1.00)		1.00(1.00, 1.00)		1.00(1.00, 1.00)	
Location	Large Urban	0.70(0.51, 0.95)	0.021	0.92(0.54, 1.58)	0.766	0.86(0.49, 1.52)	0.604
	Medium Urban	0.95(0.65, 1.38)	0.778	1.50(0.81, 2.79)	0.199	1.35(0.75, 2.41)	0.319
	Small/Rural (REF)	1.00(1.00, 1.00)		1.00(1.00, 1.00)		1.00(1.00, 1.00)	
BMI	Not Stated	0.88(0.70, 1.10)	0.250	1.23(0.83, 1.83)	0.306	1.01(0.75, 1.36)	0.956
	Obese	1.15(0.84, 1.57)	0.393	0.75(0.33, 1.68)	0.482	0.89(0.51, 1.54)	0.666
	Overweight	0.94(0.75, 1.17)	0.553	0.69(0.34, 1.38)	0.290	1.10(0.73, 1.66)	0.659
	Underweight	1.01(0.52, 1.96)	0.979	0.62(0.08, 4.59)	0.641	0.37(0.05, 2.66)	0.323
	Healthy Weight (REF)	1.00(1.00, 1.00)		1.00(1.00, 1.00)		1.00(1.00, 1.00)	
Mean SSB		1.00(0.95, 1.05)	0.948	1.05(0.94, 1.16)	0.392	1.02(0.93, 1.11)	0.707
Mean HED		1.24(1.13, 1.37)	<0.001	1.08(0.84, 1.40)	0.531	1.40(1.23, 1.59)	<0.001
Mean CTWS		1.10(1.05, 1.15)	<0.001	1.05(0.93, 1.18)	0.416	1.21(1.12, 1.30)	<0.001
Mean CTOS		1.01(0.95, 1.08)	0.685	1.04(0.91, 1.19)	0.549	1.10(0.99, 1.22)	0.086
Difference SSB		1.00(0.96, 1.04)	0.971	1.06(0.98, 1.14)	0.129	1.02(0.96, 1.09)	0.479
Difference HED		1.23(1.14, 1.33)	<0.001	1.26(1.13, 1.40)	<0.001	1.34(1.21, 1.48)	<0.001

Difference CTWS	1.08(1.05, 1.12)	<0.001	1.05(0.96, 1.14)	0.291	1.15(1.10, 1.21)	<0.001
Difference CTOS	1.03(0.98, 1.08)	0.254	1.10(1.01, 1.19)	0.022	1.04(0.96, 1.13)	0.375

Note. SSB= Sugar-sweetened beverages. CTWS = Coffee and tea with sugar. CTOS = Coffee and tea without sugar. HED= High energy drinks. OR= Odds ratio. CI = 95% Confidence Intervals. BMI= Body mass index. No-Yes= not vaping at time 1 but vaping at time 2. Yes-no = Vaping at time 1 and not vaping at time 2. Yes-Yes = Vaping at both time 1 and time 2. No-no = not vaping at either time point and is referent. REF = referent.

Table S3. Model 3: Longitudinal association and covariate effects between change in beverage consumption (2016/17-2017/2018) and never smokers, current smokers and former smokers at 2017/2018 in the COMPASS study

		**Current Smoker**		**Former Smoker**	
Category	Level	OR (CI)	*p*	OR (CI)	*p*
Gender	Male	1.42(0.94, 2.14)	0.092	3.40(1.11, 10.40)	0.032
	Female (REF)	1.00(1.00, 1.00)		1.00(1.00, 1.00)	
Grade	10	5603234(463.40, 6.775E10)	0.001	1778680(1778680, 1778680)	^
	11	2404586(490.05, 1.18E10)	<0.001	1336538(1336538, 1336538)	^
	12	348225.8(56.14,2.1601E9)	0.004	0.75(0.75, 0.75)	^
	9 (REF)	1.00(1.00, 1.00)		1.00(1.00, 1.00)	
Race	Black	1.80(0.81, 4.01)	0.152	0.00(0.00, 0.00)	0
	Asian	1.20(0.49, 2.92)	0.694	0.00(0.00, 0.00)	0
	Hispanic	1.94(0.52, 7.17)	0.323	3.30(0.46, 23.51)	0.233
	Other/Mixed/Missing	1.45(0.84, 2.50)	0.180	1.39(0.42, 4.66)	0.590
	White (REF)	1.00(1.00, 1.00)		1.00(1.00, 1.00)	
School Median Income	50001-75000	1.15(0.45, 2.97)	0.772	0.11(0.01, 1.63)	0.108
	75000-100000	1.82(0.71, 4.66)	0.211	0.16(0.02, 1.59)	0.117
	>100000	0.81(0.22, 2.93)	0.744	0.21(0.01, 3.34)	0.271
	25001-50000	1.00(1.00, 1.00)		1.00(1.00, 1.00)	
Location	Large Urban	0.41(0.25, 0.70)	<0.001	0.06(0.01, 0.50)	0.009
	Medium Urban	1.22(0.75, 1.99)	0.424	0.59(0.17, 2.03)	0.403
	Small/Rural (REF)	1.00(1.00, 1.00)		1.00(1.00, 1.00)	
BMI	Not Stated	1.47(0.88, 2.43)	0.138	0.64(0.18, 2.29)	0.489
	Obese	1.55(0.77, 3.11)	0.217	0.48(0.05, 4.58)	0.524
	Overweight	1.07(0.61, 1.88)	0.821	0.83(0.19, 3.63)	0.807
	Underweight	0.81(0.11, 6.08)	0.836	0.00(0.00, 0.00)	0
	Healthy Weight (REF)	1.00(1.00, 1.00)		1.00(1.00, 1.00)	
Mean SSB		1.06(0.95, 1.19)	0.287	0.95(0.67, 1.33)	0.744
Mean HED		1.95(1.71, 2.23)	<0.001	1.66(1.07, 2.59)	0.025
Mean CTWS		1.26(1.15, 1.38)	<0.001	1.38(1.03, 1.84)	0.031
Mean CTOS		1.02(0.88, 1.19)	0.765	1.10(0.77, 1.58)	0.594

Difference SSB	1.01(0.87, 1.17)	0.943	1.13(0.67, 1.91)	0.647
Difference HED	1.13(0.92, 1.38)	0.230	0.58(0.36, 0.92)	0.021
Difference CTWS	0.92(0.81, 1.05)	0.232	0.88(0.70, 1.11)	0.272
Difference CTOS	0.91(0.77, 1.07)	0.260	1.05(0.72, 1.54)	0.785

Note. SSB= Sugar-sweetened beverages. CTWS = Coffee and tea with sugar. CTOS = Coffee and tea without sugar. HED= High energy drinks. OR= Odds ratio. CI = 95% Confidence Intervals. BMI= Body mass index. Never smoker is referent. REF= Referent. ^ = *p* value cannot be computed.

Table S4. Model 4: Longitudinal association and covariate effects between change in beverage consumption (2016/17-2017/2018) and current vaping status at Time 3 (2017/2018) in the COMPASS study

		**Current Vaper**	
Category	Level	OR (CI)	*p*
Gender	Male	1.91(1.64, 2.21)	<0.001
	Female (REF)	1.00(1.00, 1.00)	
Grade	10	0.88(0.22, 3.49)	0.850
	11	2.14(0.56, 8.24)	0.269
	12	2.69(0.67, 10.76)	0.162
	9 (REF)	1.00(1.00, 1.00)	
Race	Black	0.72(0.45, 1.15)	0.165
	Asian	0.55(0.43, 0.70)	<0.001
	Hispanic	0.68(0.48, 0.98)	0.037
	Other/Mixed/Missing	0.82(0.68, 0.98)	0.028
	White (REF)	1.00(1.00, 1.00)	
School Median Income	50001-75000	1.29(0.81, 2.05)	0.293
	75000-100000	1.12(0.76, 1.65)	0.555
	>100000	1.01(0.62, 1.65)	0.954
	25001-50000	1.00(1.00, 1.00)	
Location	Large Urban	0.74(0.53, 1.04)	0.087
	Medium Urban	1.08(0.74, 1.56)	0.692
	Small/Rural (REF)	1.00(1.00, 1.00)	
BMI	Not Stated	0.90(0.78, 1.04)	0.169
	Obese	0.90(0.72, 1.14)	0.390
	Overweight	0.95(0.77, 1.18)	0.636
	Underweight	0.79(0.49, 1.29)	0.353
	Healthy Weight (REF)	1.00(1.00, 1.00)	
Mean SSB		1.03(1.00, 1.07)	0.060
Mean HED		1.40(1.31, 1.49)	<0.001
Mean CTWS		1.10(1.06, 1.14)	<0.001

Mean CTOS	1.01(0.97, 1.06)	0.524
Difference SSB	0.96(0.92, 1.01)	0.128
Difference HED	1.06(0.98, 1.15)	0.121
Difference CTWS	1.07(1.03, 1.12)	<0.001
Difference CTOS	1.08(1.02, 1.14)	0.009

Note. SSB= Sugar-sweetened beverages. CTWS = Coffee and tea with sugar. CTOS = Coffee and tea without sugar. HED= High energy drinks. OR= Odds ratio. CI = 95% Confidence Intervals. BMI= Body mass index. Yes= Currently vaping at time 2. No = not vaping at time 2 and is referent. REF = referent.

Table S5. Descriptive statistics of beverage consumption at all times and smoking at all time points for linked data in the study

Variable	Time C Mean	Time C SD	Time 1 Mean	Time 1 SD	Time 2 Mean	Time 2 SD
Days Drinking High energy drink	2.44	2.10	2.30	2.12	2.21	2.17
Days drinking sugar-sweetened beverages	0.19	0.76	0.24	0.91	0.30	1.04
Days drinking coffee/tea with sugar	1.57	2.11	1.78	2.24	2.01	2.38
Day drinking coffee/tea without sugar	0.68	1.66	0.76	1.75	0.94	1.97

Note. SD= Standard deviation. Time C= Time used to control for previous smoking and vaping in our analysis.

Table S6. Frequency and percent of beverage consumption at all times and smoking at all time points for entire linked sample in the study

Variable	Time C: %(N)	Time 1: %(N)	Time 2: %(N)
Have you ever smoked 100 or more whole cigarettes in your life	1.0 (61)	2.7 (160)	5.5 (321)
On how many of the last 30 days did you smoke one or more cigarettes?			
None	7.0 (416)	11.4 (679)	15.8 (933)
1 day	1.1 (64)	2.2 (129)	3.2 (191)
2-3 days	0.7 (41)	1.1 (67)	2.1 (122)
4-5 days	0.3 (17)	0.5 (32)	0.9 (55)
6-10 days	0.1 (8)	0.5 (29)	0.8 (47)
11-20 days	0.2 (13)	0.4 (21)	0.7 (42)
21-29 days	0.2 (9)	0.4 (26)	0.8 (45)
30 days	0.4 (26)	1.0 (62)	2.0 (120)
Question not asked	90.0 (5335)	82.2 (4892)	73.5 (4335)
Not stated	0	0.2 (13)	0.1 (5)
Have you ever tried an electronic cigarette, also known as an e-cigarette?	16.4 (949)	24.6 (1440)	37.5 (2198)
On how many of the last 30 days have you used an e-cigarette?			
None	93.4 (5536)	89.7 (5339)	77.0 (4542)
1 day	2.5 (148)	3.7 (218)	6.8 (400)

2-3 days	1.0 (57)	2.2 (128)	5.2 (306)
4-5 days	0.4 (22)	1.1 (65)	2.5 (150)
6-10 days	0.5 (31)	0.6 (37)	2.2 (127)
11-20 days	0.2 (10)	0.4 (21)	2.1 (124)
21-29 days	0.1 (6)	0.3 (15)	1.2 (71)
30 days	0.1 (7)	0.6 (38)	2.5 (148)
Question not asked	1.2 (69)	0.5 (29)	0
Not stated	0.7 (43)	1.0 (60)	0.5 (27)

Note. N = number of students in the sample. % = percent of students in the sample. Time C= Time used to control for smoking and vaping in our analysis.
